# 680. Comparison of Extended Duration Vancomycin Regimens for *Clostridioides difficile* Infection in Patients on Prolonged Antibiotic Courses

**DOI:** 10.1093/ofid/ofad500.742

**Published:** 2023-11-27

**Authors:** Christopher Garzia, Angela Loo, Christine J Kubin

**Affiliations:** NewYork-Presbyterian Hospital, New York, New York; New York-Presbyterian Hospital, New York, New York; NewYork-Presbyterian Hospital, New York, New York

## Abstract

**Background:**

Antibiotic exposure is a primary risk factor for *Clostridioides difficile* infection (CDI) and recurrence. We compared CDI recurrence in patients who received extended oral vancomycin (poV) after diagnosis of CDI, at a dose of q12-24h compared to q6h, while on concomitant systemic antibiotic therapy.

**Methods:**

Retrospective study of adult patients admitted 2/2020-9/2022 to NewYork-Presbyterian Hospital, diagnosed with CDI, and received at least 14 days poV including initial CDI therapy, and at least 2 days of extension with concomitant systemic antibiotics with no interruptions. Patients were grouped based on vancomycin extended dosing to either q6h or q12-24h. Patients were excluded if CDI regimen included vancomycin pulse/taper or fidaxomicin, >1 day interruption between final day of CDI treatment and extended dosing, and incomplete resolution of CDI at day 14. The primary outcome of recurrent CDI at 90 days was compared between the two groups via chi-squared test. Predictors of CDI recurrence were assessed using a logistic regression model.

**Results:**

110 patients were included:77 received q6h and 33 received q12-24h extended poV. Median age was 63 years (IQR 55,74), 51% were hospital onset-CDI, and the median total duration of poV was 25 days (IQR 19,38 days). The only significant differences between q6h and q12-24h groups were total days of vancomycin (22 vs. 37 days; p< 0.001) and patients with previous CDI episodes (7% vs 24%; p=0.02). Recurrence at 90 days was similar between q6h and q12-24h groups (13.2% and 6.3% respectively; p=0.505). On multivariable analysis, while controlling for severe and fulminant CDI and patients with inflammatory bowel disease, poV regimen of q12h-24h did not impact recurrence (OR 0.428, 95% CI 0.08-2.21).

**Conclusion:**

No significant differences were seen in CDI recurrence between extended poV q6h compared to q12-24h. For patients who require extended poV dosing beyond an initial treatment course, q12h-24h dosing may be an option with improved patient convenience and reduced drug cost. The long-term impact of this reduced dosing frequency remains to be determined.

**Disclosures:**

**All Authors**: No reported disclosures

